# Managing Complete Concentric Collapse in Obstructive Sleep Apnea: A Narrative Review

**DOI:** 10.7759/cureus.91910

**Published:** 2025-09-09

**Authors:** Kalin R Sorenson, Abigail Fish, Benjamin D Brooks

**Affiliations:** 1 Otolaryngology, Rocky Vista University College of Osteopathic Medicine, Ivins, USA; 2 Otolaryngology-Head and Neck Surgery, University of Pittsburgh Medical Center Hamot, Erie, USA; 3 Biomedical Sciences, Rocky Vista University College of Osteopathic Medicine, Ivins, USA

**Keywords:** bilateral hypoglossal nerve stimulation, complete concentric collapse, concentric collapse, failure of cpap, genio device, inspire device, levanova device, obstructive sleep apnoea, unilateral hypoglossal nerve stimulation, uvulopalatopharyngoplasty

## Abstract

Obstructive sleep apnea (OSA) is a prevalent disorder characterized by intermittent airway obstruction during sleep, often leading to significant cardiovascular and metabolic consequences. One particularly challenging type of OSA involves complete concentric collapse (CCC) of the upper airway, which complicates management by limiting the effectiveness of conventional therapies such as continuous positive airway pressure (CPAP) and hypoglossal nerve stimulation (HGNS). This narrative review examines current surgical treatments for OSA, including uvulopalatopharyngoplasty (UPPP), expansion sphincter pharyngoplasty (ESP), maxillomandibular advancement (MMA), and other skeletal surgeries, with a focus on the challenges of treating patients with CCC. We discuss the limitations of HGNS in this population and critically review studies that have contraindicated HGNS due to airway dynamics, highlighting the small patient populations and limited generalizability. Finally, we explore the potential of bilateral HGNS (B-HGNS) as a promising alternative, which may synchronize the activation of both genioglossus muscles and improve airway stability. This review emphasizes the need for further research on B-HGNS and the development of treatment strategies that reduce reliance on invasive surgeries and optimize outcomes for patients with CCC.

## Introduction and background

Obstructive sleep apnea (OSA) is a common disorder marked by repeated airway obstruction during sleep, causing oxygen desaturation, fragmented sleep, and daytime symptoms such as fatigue and cognitive impairment. Risk factors include obesity, older age, male sex, and craniofacial anatomy. Untreated OSA increases the risk of cardiovascular and metabolic disease as well as all-cause mortality [1]. Treatment options range from lifestyle changes, weight loss, and oral appliances to surgical procedures. Continuous positive airway pressure (CPAP) remains the standard of care, reliably reducing the Apnea-Hypopnea Index (AHI; number of apneas and hypopneas per hour of sleep). However, poor adherence due to discomfort or inconvenience has driven the development of alternatives such as hypoglossal nerve stimulation (HGNS), which stabilizes the airway by activating tongue protrusion muscles [2,3].

Despite the success of HGNS in OSA, not all patients can benefit from it. A specific collapse pattern, complete concentric collapse (CCC) of the soft palate, is a contraindication because tongue-based stimulation does not address circumferential palatal obstruction. This exclusion highlights an important treatment gap for patients who cannot tolerate CPAP or benefit from existing surgical options. This narrative review examines CCC in the context of OSA, including its physiological mechanisms, its impact on patient candidacy for HGNS, and current surgical approaches that have been applied to manage this collapse pattern. It also reviews emerging therapies, such as bilateral HGNS, that may offer new solutions for this challenging patient population.

To identify relevant literature, a search was conducted in PubMed and Google Scholar (November 2024-May 2025) using terms such as “obstructive sleep apnea,” “complete concentric collapse,” “CPAP failure,” “hypoglossal nerve stimulation,” and related surgical and neuromodulation treatments. English-language studies were included if they addressed CCC pathophysiology or treatment outcomes. Emphasis was placed on peer-reviewed clinical studies, large cohorts, and professional guidelines; case reports were included when they provided novel insights. Pediatric-focused studies and those without treatment relevance were excluded. No meta-analysis or pooled synthesis was performed, as this review is descriptive in nature.

## Review

Complete concentric collapse

CCC is a distinct pattern of upper airway obstruction identified during Drug-Induced Sleep Endoscopy (DISE) in patients with OSA. It is characterized by a circumferential inward collapse occurring at the level of the velopharynx, or soft palate region, which differs from the more commonly observed anteroposterior or lateral collapse of the soft palate [4]. In addition to the soft palate, other anatomical structures may contribute to CCC, including the lateral pharyngeal walls and the pharyngeal muscles, specifically the tensor and levator veli palatini, palatoglossus, and palatopharyngeus. The lateral pharyngeal walls play a crucial role in maintaining airway stability and patency, and their involvement in CCC may exacerbate airway narrowing. Similarly, dysfunction of the pharyngeal musculature or anatomical predispositions such as excess tissue or reduced muscle tone can increase the likelihood of CCC by compromising airway integrity. Additional contributors to pharyngeal collapsibility include increased nasal resistance, loss of nasal afferents, mouth breathing and mouth opening, as well as factors such as anatomic configuration, lymphoid hypertrophy, pharyngeal muscle tone, tracheal traction, weight, age, and arousal state [5].

A significant concern regarding CCC is its strong association with poor responsiveness to unilateral HGNS. This association is primarily based on a single study by Vanderveken et al. (2013; n = 17), which demonstrated suboptimal outcomes in patients with CCC, though the small sample size limits generalizability to the broader CCC population and will be further discussed later. Due to these findings, the presence of CCC has become an absolute contraindication for HGNS, highlighting the importance of thorough preoperative evaluation, including DISE, to optimize patient selection and identify other collapse patterns associated with reduced treatment efficacy [6].

Diagnosis of obstructive sleep apnea 

**Figure 1:**
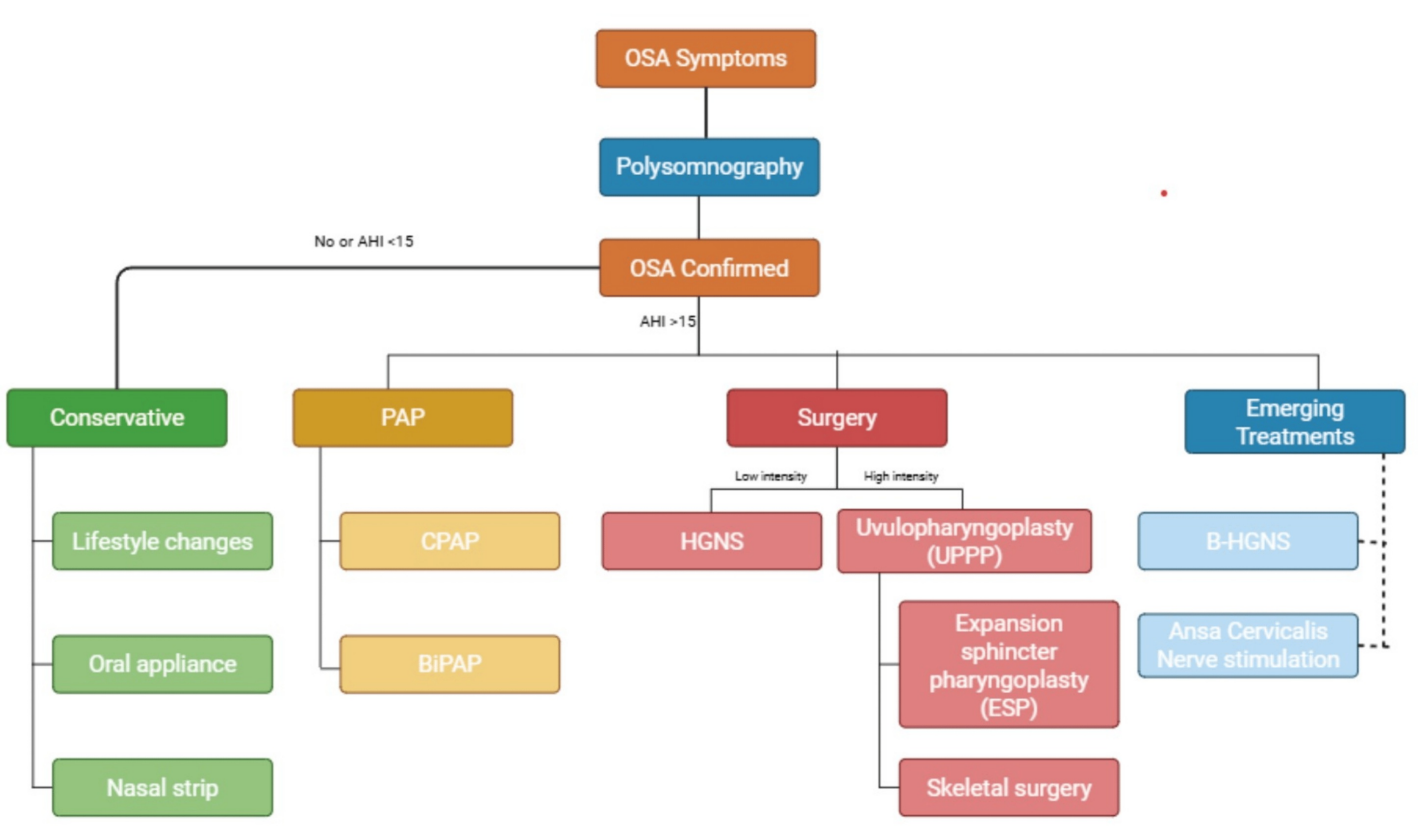
Flowchart depicting the standard of care for patients with obstructive sleep apnea, as well as current and emerging treatments. Figure created using BioRender.com with a licensed academic account.

OSA is diagnosed primarily through polysomnography (PSG), a comprehensive sleep study that measures various physiological parameters during sleep. This diagnostic tool evaluates brain activity, eye movements, heart rate, respiratory effort, airflow, oxygen saturation, and limb movements. PSG is considered the gold standard for diagnosing OSA due to its ability to provide a detailed assessment of sleep architecture and apnea-hypopnea events (Figure 1). Based on the AHI in adult patients, the severity of OSA is classified as mild (5-14 events/hour), moderate (15-30 events/hour), or severe (more than 30 events/hour) [7].

The AHI reflects the number of apneas (complete cessation of airflow) and hypopneas (partial airway obstruction resulting in decreased oxygen saturation) per hour of sleep. This metric, while critical, does not capture the full complexity of OSA on an anatomic level. Complementary metrics, such as the oxygen desaturation index (ODI, the number of times per hour of sleep that blood oxygen levels drop by a certain degree from baseline) and the arousal index, are often used to provide a more nuanced understanding of the disorder's impact on the patient's health.

For select patients, home sleep apnea testing (HSAT) may be utilized as an alternative to in-laboratory PSG. HSAT is less resource-intensive and more convenient for patients but is generally reserved for individuals with a high pretest probability of moderate to severe OSA and without significant comorbidities or other sleep disorders. Unlike PSG, HSAT typically measures a limited set of parameters, such as airflow, respiratory effort, and oxygen saturation, and may not provide information on sleep stages or arousals. Consequently, HSAT has limitations in diagnosing mild OSA or identifying other sleep-related conditions, such as periodic limb movement disorder or central sleep apnea. Clinical guidelines from the American Academy of Sleep Medicine (Table 1) also recommend important indications for follow-up PSG and HSAT in adult patients with OSA [8].

**Table 1:** Clinical guidelines from the American Academy of Sleep Medicine on important indications for follow-up PSG and HSAT in adult patients with OSA Adapted from Reference no. [8]. PSG = Polysomnography; HSAT = Home Sleep Apnea Test; OSA = Obstructive Sleep Apnea; PAP = Positive Airway Pressure.

Indication	Follow-up Test (PSG or HSAT)	Recommendation
Routine reassessment of asymptomatic patients on PAP	PSG or HSAT	Not recommended
Recurrent or persistent symptoms despite good PAP adherence	PSG or HSAT	May be used
Non-PAP interventions (e.g., oral appliances, surgery)	PSG or HSAT	Recommended
Clinically significant weight change (gain or loss)	PSG or HSAT	May be used
Reassessment of sleep-related hypoxemia/hypoventilation after OSA treatment	PSG	May be used
Development or change in cardiovascular disease in a treated OSA patient	PSG or HSAT	May be used
Unexplained PAP device-generated data	PSG	May be used

Although PSG and HSAT are essential for establishing the presence and severity of OSA, they offer limited insight into the anatomic characteristics of airway obstruction. CCC, in particular, cannot be accurately distinguished by sleep study metrics alone. To address this gap, drug-induced sleep endoscopy (DISE) has emerged as the cornerstone of CCC evaluation, providing dynamic, real-time visualization of airway collapse patterns that inform patient selection and therapeutic planning.

Drug-induced sleep endoscopy

DISE is a widely utilized upper airway evaluation technique that provides a dynamic, real-time assessment of airway collapse in patients with OSA. This procedure involves the use of a flexible fiberoptic endoscope to directly visualize the upper airway while the patient is under controlled conditions of unconscious sedation. By simulating sleep, DISE allows for the identification of specific patterns and sites of airway obstruction, offering a comprehensive three-dimensional examination of airway dynamics [4]. The procedure is typically performed in a controlled clinical setting, often in an operating room or endoscopy suite, where sedation is carefully titrated to achieve a sleep state that mimics natural sleep as closely as possible. Various sedative agents, such as propofol, dexmedetomidine, or midazolam, may be used, though there is no universal consensus on the optimal choice of medication. Once the patient reaches an appropriate level of sedation, the endoscope is inserted transnasally to assess the velum (soft palate), oropharynx, tongue base, and epiglottis, commonly categorized using the VOTE classification system. Because DISE can reveal a wide range of collapse patterns, the Velum Oropharynx Tongue base Epiglottis (VOTE) classification system is commonly used to provide a structured, reproducible framework for evaluating obstruction at four anatomical levels (Table 2).

**Table 2:** This table provides information about the VOTE classification system used to determine collapse patterns during DISE Adapted from Reference no. [4]. CCC = Complete Concentric Collapse, VOTE = Velum Oropharynx Tongue base Epiglottis, DISE = Drug-Induced Sleep Endoscopy.

Region	Anatomical Focus	Collapse Pattern(s)
Velum (V)	Soft palate and velopharyngeal region	Anteroposterior, lateral, or concentric (CCC)
Oropharynx (O)	Lateral pharyngeal walls	Typically lateral; contributes to overall airway obstruction
Tongue Base (T)	Base of the tongue	Anteroposterior collapse; impinges on the airway
Epiglottis (E)	Epiglottis	Retroflexed (posterior) or lateral collapse

**Figure 2:**
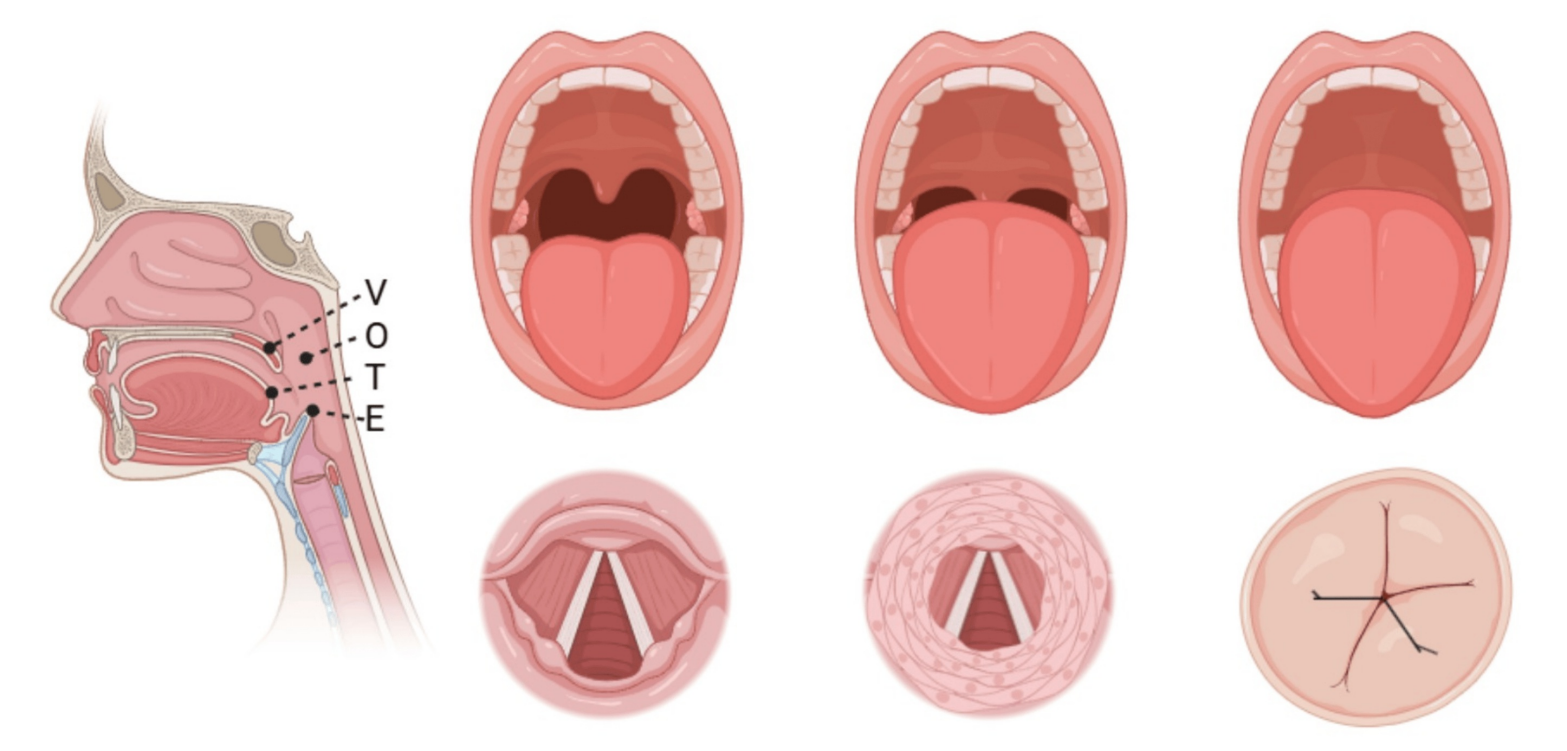
This figure demonstrates the different locations obstruction can occur as it relates to the VOTE classification Figure created using BioRender.com with a licensed academic account. VOTE = Velum Oropharynx Tongue base Epiglottis.

Each anatomical region is graded based on the degree of collapse observed during assessment (Figure 2). Collapse is categorized as no collapse (0%), partial collapse (50%), or complete collapse (100%).

The VOTE classification provides a structured framework to describe airway obstruction during DISE and plays a critical role in guiding treatment decisions, including candidacy for HGNS [4]. Despite its advantages, DISE has notable limitations. One key concern is its inconsistent inter-rater reliability, as different clinicians may interpret findings variably. Additionally, questions remain regarding how well DISE truly reflects airway dynamics during natural sleep, given the potential pharmacologic effects of sedation on neuromuscular tone [9,10]. Furthermore, there is no universally accepted grading system for quantifying airway collapse, and variation in sedative choice may influence results. Nevertheless, DISE remains a cornerstone of preoperative evaluation for HGNS, as it provides valuable insight into airway anatomy and collapse patterns, which are critical for selecting appropriate candidates for this therapy [6].

Conventional surgical therapies 

The gold standard treatment for OSA is CPAP therapy, which uses a mask to deliver pressurized air, keeping the airway open during sleep. CPAP effectively reduces AHI, improves sleep quality, and lowers blood pressure. However, patient compliance remains a significant barrier, with studies reporting nonadherence rates ranging from 29% to 84% due to factors such as discomfort, claustrophobia, and nasal obstruction [2]. Here, we will discuss some surgical treatments and study outcomes for effectiveness.

Uvulopalatopharyngoplasty (UPPP)

UPPP is the most commonly performed surgical treatment for OSA, typically recommended for patients who have not responded to medical therapy or are unable to tolerate CPAP. It involves removing the uvula, the soft palate's distal margin, palatine tonsils, and excess tissue from the lateral pharynx. Success is influenced by anatomical factors, particularly the location of airway collapse, with patients having isolated velopharyngeal obstruction and lower BMI more likely to achieve favorable outcomes [11-13]. Historical success rates vary between 40% and 66% depending on patient selection.

Long-term studies reinforce this variability. A retrospective Mayo Clinic review of 63 patients reported that younger age, lower BMI, and less severe OSA were predictors of better outcomes, but also highlighted limitations such as selection bias and the difficulty of capturing late complications [14]. Other reports have shown that even patients initially deemed surgical “successes” can relapse over time, underscoring the importance of objective follow-up [15]. Modified UPPP without tonsillectomy (UPsT) can be considered in patients with small tonsils and isolated velopharyngeal collapse, although outcomes are poorer in the presence of CCC [16,17]. These procedures demonstrate that careful selection and preoperative assessment, such as DISE, are critical for surgical success (see Table 3 for a summary of surgical outcomes).

**Table 3:** Summary of surgical therapies for obstructive sleep apnea This table provides an overview of commonly performed surgical procedures for obstructive sleep apnea (OSA), including patient selection, reported outcomes, invasiveness and risk, relevance to complete concentric collapse (CCC), and key references. Palatal surgeries (uvulopalatopharyngoplasty (UPPP), modified UPPP without tonsillectomy (UPsT), expansion sphincter pharyngoplasty (ESP)) show variable success influenced by anatomy and BMI, with ESP uniquely capable of converting CCC to an anterior–posterior collapse pattern, enabling eligibility for HGNS. Skeletal surgeries (MMA, hyoid suspension) are highly effective but carry higher invasiveness and complication risks, and are typically reserved for severe or multilevel OSA. Data are drawn from the cited studies and highlight surgical success rates and candidacy for specific procedures in patients with CCC. AHI = Apnea-Hypopnea Index.

Surgical Procedure	Patient Selection / Notes	AHI Reduction / Success	Invasiveness / Risk	CCC Relevance	References
UPPP	Isolated velopharyngeal obstruction, lower BMI	40–66% achieved ≥50% AHI reduction	Moderate	CCC associated with poorer outcomes	[11–13]
UPsT (UPPP without tonsillectomy)	Small tonsils, isolated velopharyngeal collapse	Loose criteria: 82% success; Strict criteria: 68%	Moderate	CCC associated with lower success rates	[16,17]
ESP	Lateral pharyngeal wall collapse, CCC at velopharynx	Converted CCC to AP collapse; combined ESP + HGNS: mean AHI dropped from severe to mild	Moderate	Converts CCC to favorable pattern, enabling HGNS	[18,19]
MMA / Hyoid Suspension	Severe or multilevel OSA, not candidates for less invasive surgery	Significant AHI reduction; among most effective surgical options	High	Effective regardless of CCC, but high invasiveness limits use	[13,18]

Expansion Sphincter Pharyngoplasty (ESP)

ESP targets lateral pharyngeal wall collapse and is used in select patients with CCC at the velopharynx. The technique basically consists of a tonsillectomy, expansion pharyngoplasty, rotation of the palatopharyngeus muscle, a partial uvulectomy, and closure of the anterior and posterior tonsillar pillars. This procedure can be performed alone or as part of the multilevel surgical algorithm in the treatment of OSA. Studies show ESP can convert CCC to an anterior-posterior collapse pattern, making patients eligible for HGNS [18]. Combined with HGNS in a two-stage approach, ESP may substantially improve outcomes. While promising, further research is needed to verify these results in broader populations and to assess the long-term safety, efficacy, and clinical utility of multilevel upper airway surgery for OSA [19].

*Skeletal Surgery* 

Skeletal surgery includes hyoid suspension and maxillomandibular advancement (MMA). Hyoid suspension involves suturing the hyoid bone to the thyroid cartilage or suspending the hyoid to screws anchored into the mandible. This technique pulls the epiglottis, base of the tongue, and soft tissues of the airway forward. MMA advances both the maxilla and mandible, creating tensions throughout the airway and decreasing tissue collapsibility. These techniques are considered to be the most invasive with the highest risk of complications, and therefore are less favorable among patients [13,18]. Skeletal surgeries such as MMA are among the most effective at enlarging the airway but are reserved for select patients given their invasiveness and higher complication risk.

In summary, CPAP remains the most reliably effective treatment for OSA but is limited by high nonadherence rates. UPPP and its modified forms can provide meaningful reductions in AHI for selected patients, particularly those with a lower BMI or isolated velopharyngeal collapse, though outcomes are less favorable in patients with CCC. ESP shows greater effectiveness for addressing CCC, especially when combined with staged HGNS, while skeletal surgeries such as MMA offer the highest likelihood of anatomical correction but carry increased procedural risk. These findings highlight that, although traditional therapies are effective for many patients, those with CCC may benefit from exploring emerging treatment interventions.

Hypoglossal nerve stimulation

Unilateral HGNS has become an established treatment option for patients with moderate to severe obstructive sleep apnea who are intolerant of CPAP. By electrically stimulating the hypoglossal nerve, HGNS activates the genioglossus muscle, leading to anterior displacement of the tongue and enlargement of the retrolingual airway. Clinical trials, including the pivotal STAR trial, have demonstrated significant reductions in AHI, improved oxygen saturation, and enhanced quality of life in appropriately selected patients [20]. Long-term outcomes from the five-year follow-up of the STAR trial confirmed the durability of these benefits, with sustained improvements in sleepiness, quality of life, and respiratory parameters [21]. However, unilateral HGNS is contraindicated in cases of CCC at the level of the soft palate, as the circumferential nature of this collapse is not adequately addressed by tongue protrusion alone. This limitation has prompted investigation into modified or bilateral stimulation strategies that may better target the unique pathophysiology of CCC.

Predictors of HGNS Success

Patient-specific anatomical and physiological factors influence HGNS efficacy. Preoperative evaluation, including DISE, is essential not only to exclude CCC but also to characterize other collapse patterns, such as anteroposterior or lateral palatal and tongue base obstruction [6]. Other predictors include baseline AHI, age, BMI, and specific sites of upper airway collapse, which can guide patient counseling and expectation management.

*Supine Pharyngeal Width* 

Weiner et al. (2022) investigated the relationship between supine pharyngeal width (SPW), CCC observed during DISE, and HGNS therapy outcomes in patients with OSA [22]. Their study aimed to determine if SPW could predict CCC and whether CCC should remain a contraindication for HGNS therapy. The researchers retrospectively analyzed a cohort of OSA patients who underwent DISE and subsequent HGNS implantation. SPW was measured in 73 patients, and treatment outcomes were assessed using standard OSA metrics such as the AHI and patient-reported improvements. The study found that patients with CCC had significantly narrower SPWs compared to those without CCC. A logistic regression model demonstrated that a narrower SPW was a significant predictor of CCC, suggesting that airway anatomy plays a role in the likelihood of CCC occurrence during DISE. For SPW >20 mm, the positive likelihood ratio for the absence of CCC was 6.67, with pre-and post-test odds of 6.3 and 42.0, respectively.

Additionally, the researchers assessed whether CCC patients had worse responses to HGNS therapy compared to non-CCC patients. Despite CCC historically being considered a poor prognostic factor, the findings revealed that CCC patients experienced comparable reductions in AHI following HGNS treatment. Postoperative PSG data were available from 31 of 44 (70.5%) patients subsequently implanted with HGNS, and those with SPW >20 mm had a greater rate of HGNS response than those with SPW ≤20 mm (62% vs. 30%; P < .05). Patient-reported outcomes, including Epworth Sleepiness Scale (ESS) scores, also improved similarly in both groups.

The study's findings have important clinical implications. Narrower SPW is significantly associated with CCC, providing a potential anatomical marker to identify patients at risk for CCC during DISE. This could help guide preoperative assessments and patient selection for HGNS therapy. Furthermore, the study challenges the historical view that CCC is an absolute contraindication for HGNS therapy. Some CCC patients achieved AHI reductions and symptomatic improvement comparable to non-CCC patients, suggesting that HGNS may still be effective for certain CCC cases. This could lead to a broader selection of patients for HGNS implantation and further investigation into modifying therapy settings for CCC patients.

In conclusion, Weiner et al. (2022) provide evidence that narrow SPW is associated with CCC and that CCC patients can still benefit from HGNS therapy [22]. These findings suggest that excluding CCC patients from HGNS treatment may be overly conservative, warranting further research into refining patient selection criteria [22]. While anatomical and physiological predictors, such as SPW, help identify patients most likely to benefit from unilateral HGNS, ongoing research is exploring alternative or adjunctive therapies, including pharmacologic interventions for weight loss and novel neuromodulation strategies, that may expand treatment options for patients with CCC or other complex collapse patterns.

Emerging therapies

GLP-1 Receptor Agonists and Weight-Loss Therapy

Beyond anatomical and neuromodulatory interventions, pharmacologic approaches targeting obesity are emerging as adjunctive therapies in OSA management, particularly given the role of weight in airway collapsibility. GLP-1 receptor agonists, such as semaglutide and tirzepatide, promote substantial weight loss and have been associated with improvements in AHI and oxygenation. Two recent meta-analyses demonstrated that GLP-1 RAs significantly reduce OSA severity and improve sleep-related outcomes, supporting their role as adjunctive therapy in selected patients [23,24]. These agents may be particularly beneficial in obesity-driven OSA or as part of a multimodal approach alongside lifestyle modification, CPAP, or HGNS. However, while these findings are encouraging for OSA broadly, no studies to date have directly examined the effects of GLP-1 RAs in patients with complete concentric collapse. Given CCC’s structural pathophysiology at the level of the soft palate, pharmacologic or weight-loss therapies may have limited success in this specific subgroup.

Bilateral Stimulation (Genio™ and aura6000™)

Building on neuromodulation strategies, novel bilateral HGNS devices are being investigated to overcome limitations of unilateral stimulation, especially in patients with CCC or complex airway collapse patterns. One study showing the effectiveness of B-HGNS showed that some patients had better opening of the soft palate, possibly due to palatoglossus coupling (PGC), linkage of muscles within the soft palate to those in the lateral tongue body by the palatoglossus muscle [25,26]. The device’s effectiveness in reducing the AHI and OSI in other countries [5,27] has led to further research for use here in the United States.

The Genio™ system consists of an implantable stimulator placed over both genioglossus muscles with paddle electrodes oriented toward the medial branches of the hypoglossal nerves. At night, patients wear a disposable activation patch under the chin that wirelessly transmits energy to the stimulator [28,29]. This bilateral stimulation improves tongue symmetry and may enhance soft palate stability by synchronizing activation of both sides of the tongue.

Preliminary studies suggest better outcomes in anatomically complex OSA patients, as it may mitigate CCC through synchronized activation of the tongue [29]. Current data of 115 patients in a United States clinical trial, labeled the DREAM study, was presented at the International Surgical Sleep Society Congress in 2024. The study results so far have shown that 82.0% of patients who underwent a polysomnography at 12 months post-operation (post-op) and had an AHI below 15, and 67.4% of patients who underwent polysomnography at 12 months post-op had an AHI below 10 [30].

The LivaNova device, the aura6000™, is also an HGNS that is being trialed in the United States in the OSPREY clinical trial. Like Genio™, it stimulates both hypoglossal nerves, but unlike the transdermal Genio system, it is a fully implantable device similar in form to Inspire™. The system consists of an implantable pulse generator (IPG) connected to bilateral stimulation leads placed along the hypoglossal nerves, as well as a sensing lead that monitors respiration in real time. Using this closed-loop feedback, the IPG delivers stimulation in synchrony with inspiration, ensuring activation of both sides of the tongue only when airway obstruction is most likely to occur. This contrasts with Genio’s synchronized paddle electrodes and external activation patch.

Current data from the OSPREY clinical trial show that AHI was reduced by 66.2% when the median at baseline of 34.3 is compared to the median of 11.6 at six months. ODI also reduced by 63.3% when the median at baseline of 34.9 is compared to the median of 12.8 at six months. Once the six-month results analysis is completed, LivaNova will submit the OSPREY clinical data to the U.S. Food and Drug Administration (FDA) as part of its premarket approval submission for the aura6000™ System [31].

Ansa Cervicalis Stimulation

Another promising avenue is stimulation of non-hypoglossal nerves, such as the ansa cervicalis, which may reduce collapsibility across multiple airway levels and complement existing therapies. A recent study showed that bilateral ACS of the sternothyroid muscle has the potential to decrease collapsibility of all airway flow-limiting structures, including the soft palate, oropharyngeal walls, tongue base, and epiglottis, and increase VImax [32]. These findings are limited to early, small-scale investigations. The long-term safety, reproducibility, and real-world clinical impact of ACS remain uncertain. Larger trials are necessary to determine whether ACS can provide meaningful improvements in OSA, particularly in patients with CCC.

Key takeaways from the literature

**Figure 3:**
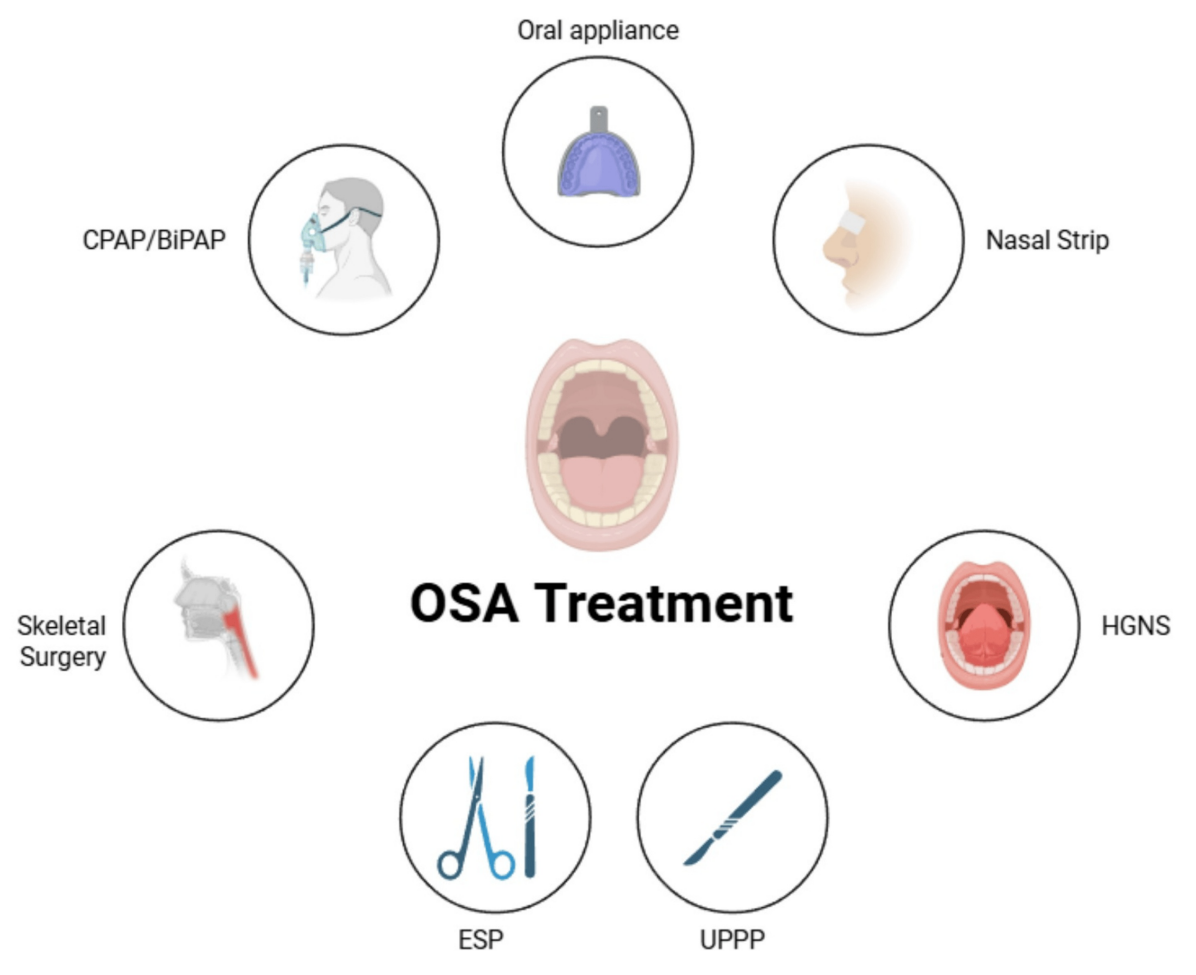
This figure broadly depicts available treatment options for obstructive sleep apnea Figure created using BioRender.com with a licensed academic account.

The treatment of OSA in patients with CCC remains one of the most challenging aspects of managing the disorder. CCC refers to the complete collapse of the velopharynx during sleep, which severely limits the ability of standard therapies (Figure 3) to maintain an open airway. Surgical interventions like UPPP, ESP, and MMA, though effective for some, may not always be suitable for CCC patients due to the complexity of their airway anatomy. These patients often require invasive and high-risk surgeries, which come with a range of potential complications and lower success rates when compared to other forms of OSA.

A significant hurdle in treating CCC is its impact on HGNS, a treatment that has been widely utilized to address OSA in many patients. HGNS works by stimulating the hypoglossal nerve to activate the tongue muscles, thereby preventing airway collapse during sleep. However, a pivotal study identified that patients with CCC were generally considered unsuitable for HGNS because the mechanism of airway collapse in these patients often involves a combination of complete concentric collapse and a lack of anterior-posterior (AP) collapse [33]. This results in the inability to generate sufficient tension in the airway walls to benefit from HGNS stimulation. The study highlighted that the lack of a dynamic airway collapse in CCC patients limits the effectiveness of HGNS, which was originally thought to primarily benefit those with AP collapse.

However, this study’s findings, while influential, also have limitations that raise concerns about the generalizability of its conclusions. A critical issue with the study was its relatively small patient population, which does not adequately represent the broader spectrum of OSA patients, particularly those with more complex airway collapse patterns. The exclusion of patients with multilevel airway obstructions, particularly those with combined CCC and AP collapse, restricts the applicability of the study’s conclusions to a wide range of individuals. This limited population undermines the argument that CCC is universally contraindicated for HGNS, and instead suggests that, with more careful patient selection and advanced diagnostic techniques like DISE, some CCC patients may still be candidates for HGNS or other innovative therapies.

Emerging therapies such as B-HGNS have the potential to address some of these limitations. Unlike traditional unilateral HGNS, which targets only one side of the tongue, bilateral stimulation synchronizes the activation of both genioglossus muscles. This approach may improve airway stability in patients with CCC by providing more balanced support across the airway. Early studies suggest that bilateral HGNS may be effective in patients who otherwise would not benefit from unilateral stimulation, offering a less invasive alternative to traditional surgeries like UPPP or MMA. In particular, bilateral stimulation could mitigate the issues associated with CCC by ensuring that both sides of the airway are reinforced during sleep.

The potential of bilateral HGNS to reduce the need for intense surgeries highlights a crucial shift in OSA management. For patients with CCC, where surgical success may be limited, bilateral HGNS represents a less invasive and more adaptable solution. It could offer an alternative for patients who would otherwise require high-risk surgeries such as MMA, which involve significant anatomical changes and extended recovery periods. Furthermore, combining B-HGNS with other less invasive therapies could lead to improved outcomes for patients with multilevel airway collapse.

While the treatment of CCC in OSA remains a complex challenge, advancements like bilateral HGNS offer hope for reducing the need for invasive surgeries and improving patient outcomes. The limitations of earlier studies on HGNS in CCC patients, due to their small sample sizes and limited patient populations, underscore the need for more comprehensive research to better understand the potential of B-HGNS in these individuals. With continued innovation and personalized treatment strategies, there is significant potential to enhance the management of OSA in patients with CCC, ultimately improving their quality of life and reducing reliance on high-risk surgical procedures.

## Conclusions

OSA is a complex disorder requiring personalized treatment. While CPAP remains the gold standard, adherence issues highlight the need for alternatives. HGNS offers a promising surgical option, but its effectiveness is limited by CCC at the velopharyngeal level. Accurate diagnostic tools, particularly DISE, are critical for identifying collapse patterns and refining patient selection. Emerging evidence suggests that even some patients with CCC, especially those with a wider supine pharyngeal width, may benefit from HGNS. The potential of bilateral stimulation to enhance tongue symmetry and mitigate airway collapse represents a promising development in the field. Surgical interventions such as UPPP and hyoid suspension, along with novel devices like Genio™ and aura6000™, further expand therapeutic options. Continued research into optimizing HGNS technology, leveraging DISE findings, and integrating multimodal approaches will be crucial to improving outcomes and quality of life in patients with OSA, especially those with challenging anatomical profiles.
